# *Editorial Commentary:* The Endgame for Serogroup A Meningococcal Disease in Africa?

**DOI:** 10.1093/cid/cis896

**Published:** 2012-10-19

**Authors:** Martin C. J. Maiden

**Affiliations:** Department of Zoology, University of Oxford, United Kingdom

**Keywords:** *Neisseria meningitides*, vaccination, herd immunity, carriage, epidemics

**(See the Major Article by Kristiansen et al on pages 354–63.)**

Since 1905 the “meningitis belt” of sub-Saharan Africa, has experienced devastating seasonal epidemics of meningococcal disease approximately every decade [1]. These occur during the Harmattan, a northerly dry wind, and, although they typically last only a matter of weeks, they cause high morbidity and mortality and have a disproportionate disruptive effect on the health care systems of countries that face numerous other challenges with scarce resources. The Meningitis Vaccine project [2], an innovative vaccine development and implementation program funded by the Bill and Melinda Gates Foundation, has recently introduced a new vaccine to this region, MenAfriVac [3], which holds the promise of ending this scourge. Kristiansen et al [4] provide important new data supporting the introduction and use of this vaccine.

The first effective meningococcal vaccines were developed in the 1960s and contained unmodified capsular polysaccharides [5], but these vaccines elicited only primary immune responses that provided short-term immunity and were ineffective in infants, a major at-risk group. Importantly for controlling disease caused by *Neisseria meningitidis*, which is transmitted asymptomatically at high rates in the population and invades comparatively rarely, these “plain” polysaccharide vaccines had little or no effect on carriage [6], and encapsulated meningococci therefore continued to circulate in vaccinated populations. These vaccines were used in Africa with some effect, but the logistics of their use were complicated, and they did not represent a permanent solution, with epidemics continuing to occur [1].

A major step forward in the control of meningococcal disease globally came with the conjugate polysaccharide vaccines, with similar products available for *Haemophilus influenzae* type b (Hib) and various pneumococcus types [7, 8]. The chemical coupling bacterial polysaccharides to a T-cell antigen, usually a protein such as diphtheria or tetanus toxoid, recruits T-cell help. After immunization with this combined antigen, an affinity-matured immune response is elicited, which is bactericidal and also confers immunological memory. These vaccines have had a major impact on disease rates of the targeted bacteria wherever they have been introduced. Although they provide excellent protection to the immunized individual, their effectiveness, largely unanticipated when they were developed, is due to the induction of herd immunity—a consequence of their effect on asymptomatic carriage [9]. The survival of any infective agent, whether disease-causing or not, is dependent on its being passed among individual hosts. The crucial property in this respect is *R*_0_, the basic reproduction number, which is the number of new infections resulting from any given infection. When the proportion of individuals in a population that are resistant to infection by a given agent—for example, as a result of immunization—is greater than 1 − (1/*R*_0_), then transmission of that agent in the population is prevented, protecting not only the immunized but also the unimmunized from the possibility of disease [10].

To achieve herd immunity it is necessary, not only to have a vaccine that is effective against carriage, but also to attain the requisite proportion of individuals resistant to infection in the cohort in which transmission occurs; this proportion is a product of vaccine efficacy and coverage. In the United Kingdom's introductions of the Hib and meningococcal C conjugate (MCC) polysaccharide vaccines, for example, this was achieved by a combination of high levels of immunization, high vaccine efficacy, and “catch-up” campaigns. The latter were originally intended to protect older individuals from disease not covered by infant administration: those <48 months old for the Hib vaccine [11] and <18 years old for the MCC vaccine [12]. However, it has subsequently been demonstrated that their major benefit was the herd immunity induced in these cohorts, where most of the transmission was occurring [13, 14]. This has important implications for the efficient use of vaccination: using these concepts, for example, The Netherlands implemented a single-dose vaccination with MCC for individuals >14 months and up to 18 years old (ie, those capable of generating an immune response that has both memory and the ability to prevent carriage), which has eliminated disease in the country even though infants are not routinely immunized [15].

As with other conjugate vaccine introductions, there were two areas of uncertainty surrounding the use of MenAfriVac: did it prevent carriage, as other conjugate polysaccharide vaccines had done, and which were the most important cohorts to immunize? As much less was known about meningococcal carriage in the meningitis belt, some of it contradictory [16], the decision was made to immunize all those under the age of 29 years and to conduct carriage surveys before and after immunization [17]. Kristiansen et al report one such survey in the first country to receive the vaccine, Burkina Faso, and the African Meningococcal Carriage Consortium (http://www.menafricar.org/) are undertaking studies across the meningitis belt.

Those monitoring the impact of meningococcal vaccines on carriage by means of point-prevalence surveys of carriage before and after the implementation of a national immunization campaigns face a number of problems related to the biology of meningococcal carriage. The meningococcus is a highly diverse organism both genetically and antigenically, with many different genotypes circulating in a given population at a given time. Only a minority of these meningococci are likely to cause disease, members of the so-called “hyperinvasive lineages” [18]; indeed, the point prevalence of these hyperinvasive lineages is often paradoxically low, considering the rates of disease which they cause. In 1999, for example, the time of the introduction of MCC vaccines in the United Kingdom, the serogroup C ST-11 strain responsible for elevated levels of disease was only 6% of the carried meningococcal population and found in only 0.3% of individuals [14]. Thus, very large surveys are required in to establish vaccine effects, with a total of 48 309 individuals sampled in the UK study. In addition, carriage rates for particular strains vary over time, possibly confounding any observations made, although these natural variations are almost certainly the major reason for the periodicity of epidemics [19].

The work of Kristiensen et al is an important contribution, because carriage surveys of a sufficient scale were completed during the vaccine introduction with isolate characterization and, importantly, appropriate quality control procedures, which are essential as these studies are challenging and require appreciable infrastructure and capacity [20]. Combined with prevaccination surveys [21] and the monitoring of meningococcal disease, which shows a dramatic reduction after vaccination [22], there is now compelling evidence for a strong herd immunity effect generated by MenAfriVac, especially because the effects were only seen in districts post-vaccination. Taken together, these data strongly suggest that the introduction of MenAfriVac, if completed as planned and maintained over time, could indeed result in the control and perhaps elimination of serogroup A meningococcal disease across the meningitis belt, which would be a further achievement for conjugate polysaccharide vaccines, arguably the unsung heroes of vaccinology of the late 20th century.

Despite this very positive prospect, however, there remain uncertainties that will have to be resolved with further research and continued vigilance. Although the effects of MCC vaccines have been sustained over at least 10 years [23], it is not known how long this effect will last with MenAfriVac. Furthermore, although capsule replacement has not yet been a major problem since the introduction of the MCC and Hib vaccines, this has been seen with the pneumococcal vaccine [24] and the presence of other meningococcal serogroups in the meningitis belt remains a concern until comprehensive vaccines can be delivered. We also need much more information on the dynamics of meningococcal carriage throughout the region, and we need to identify the cohorts and behaviors that drive transmission, enabling better targeting of vaccination efforts. The conjugate vaccines have been very successful because of interactions between immunology and bacterial population biology that became apparent only after the introduction of these vaccines; to assure their future success, it is essential that we continue to improve our understanding of these beneficial effects.
